# Mosquito community composition in South Africa and some neighboring countries

**DOI:** 10.1186/s13071-018-2824-6

**Published:** 2018-06-01

**Authors:** Anthony J. Cornel, Yoosook Lee, António Paulo Gouveia Almeida, Todd Johnson, Joel Mouatcho, Marietjie Venter, Christiaan de Jager, Leo Braack

**Affiliations:** 1 Department of Entomology & Nematology, University of California, Davis, USA; 2 UP Institute for Sustainable Malaria Control & MRC Collaborating Centre for Malaria Research, Faculty of Health Sciences, University of Pretoria, Pretoria, South Africa; 3 Global Health and Tropical Medicine, GHTM, Institute for Hygiene and Tropical Medicine, IHMT, Universidade Nova de Lisboa, UNL, Lisbon, Portugal; 4 Centre for Viral Zoonoses, Faculty of Health Sciences, University of Pretoria, Pretoria, South Africa

**Keywords:** Mosquitoes, Vectors, Arboviruses, Malaria, Shannon index, Diversity measures, Mosquito community composition

## Abstract

**Background:**

A century of studies have described particular aspects of relatively few mosquito species in southern Africa, mostly those species involved with disease transmission, specifically malaria and arboviruses. Patterns of community composition such as mosquito abundance and species diversity are often useful measures for medical entomologists to guide broader insights and projections regarding disease dynamics and potential introduction, spread or maintenance of globally spreading pathogens. However, little research has addressed these indicators in southern Africa.

**Results:**

We collected 7882 mosquitoes from net and light traps at 11 localities comprising 66 species in 8 genera. We collected an additional 8 species using supplementary collection techniques such as larval sampling, sweep-netting and indoor pyrethrum knockdown catches. Highest diversity and species richness was found in the Okavango Delta of Botswana and in South Africa’s Kruger National Park, while the lowest diversity and abundances were in the extreme southern tip of South Africa and in semi-desert Kalahari close to the South Africa border with Botswana. Species composition was more similar between proximal localities than distant ones (Linear model *P*-value = 0.005). Multiple arbovirus vector species were detected in all localities we surveyed (proportion of vector mosquito numbers were > 0.5 in all locations except Shingwedzi). Their proportions were highest (> 90%) in Vilankulo and Kogelberg.

**Conclusions:**

Multiple known arbovirus vector species were found in all study sites, whereas anopheline human malaria vector species in only some sites. The combination of net traps and light traps effectively sampled mosquito species attracted to carbon-dioxide or light, accounting for 89% of the 74 species collected. The 11% remaining species were collected using supplementary collection techniques mentioned above. The diversity of species weas highest in savanna type habitats, whereas low diversities were found in the drier Kalahari sands regions and the southern Cape fynbos regions.

**Electronic supplementary material:**

The online version of this article (10.1186/s13071-018-2824-6) contains supplementary material, which is available to authorized users.

## Background

Historic interest in the mosquitoes of southern Africa has largely been based on their role as vectors of human or animal disease. Malaria-associated morbidity and mortality was very high during the early decades of the 20th century [1–5] and gave rise to a disproportionately large body of still-increasing literature on anopheline mosquitoes [6–13]. Outbreaks of arboviral disease, associated with high mortality in livestock, resulted in sustained research on non-anopheline mosquitoes [14–22], with an associated surge in publications relating to regional surveys commencing in the 1950’s and 1960’s for arboviruses affecting humans [23–29]. In more recent decades the rapid spread and increasing global challenge posed by mosquito-borne viruses, such as West Nile, Zika and others, spurred further publications on mosquitoes [30–33].

The overwhelming majority of the mosquito literature for southern Africa addresses mainly aspects of identification, taxonomy or classification [34–40], vector potential or status in one way or another [14, 31, 41–46], or discussion of specific species in relation to insecticide resistance or other aspects of disease control [47–53]. Aside from the breeding biology of some species [54–57], little work has been done on the general ecology and compositions of mosquito communities in southern Africa. Much of the earlier knowledge is captured in summary overviews in the standard reference volumes on anophelines [58, 59] and culicines [60]. Broadly speaking, the species composition and geographical distribution patterns of anopheline mosquitoes in southern Africa are better documented than for culicines, whilst abundance trends and diversity patterns for all mosquito groups have been largely neglected or undocumented.

The increase in frequency of arbovirus outbreaks and rapid spread of such diseases, as well as scale of the public health consequences [61–65], have given rise to multiple calls for countries globally to raise vigilance regarding arboviruses [66, 67] and an associated need to understand the population status of known or potential vector mosquitoes. This paper provides an initial assessment for understanding broad patterns of mosquito diversity, abundance and distribution in southern Africa. Most of the southern African landscape has been altered, due to human agricultural and settlement influences, but a few pockets of more pristine mosquito diversity attributes should still be found in designated National Parks and wilderness areas. For this study, priority was given to natural reserves so that mosquito catches would represent the historic ‘natural’ state of populations. Therefore our results would represent baseline species diversity data which future surveys could be compared to for assessing human impact in nearby areas of land use change. These studies are also broadly aimed to develop projections and models of where arboviruses are likely to establish and persist when mosquito vector and vertebrate host data are combined.

## Methods

We limited the species diversity comparisons to one season to avoid inter-annual fluctuations by undertaking all the surveys within eight weeks, from multiple habitats, using predominantly net (CO_2_ baited) and (white light + CO_2_ baited) CDC traps [68].

### Collection period

Our surveys ran from late-January until early-April 2015, averaging three to four nights per locality. Much of the southern African region experiences summer rainfall from November to April [69]. Mosquito breeding also peaks during these hotter and wet months, so that most mosquito populations are at their highest levels from about January to mid-April. The Kogelberg Nature Reserve in the Western Cape is the exception, falling within a winter rainfall region. However, all the trapping locations in this Reserve were close to the Palmiet River and its fringing fynbos vegetation, which would be the primary source of mosquitoes independent of rain.

### Geographical distribution of study sites

**Fig. 1:**
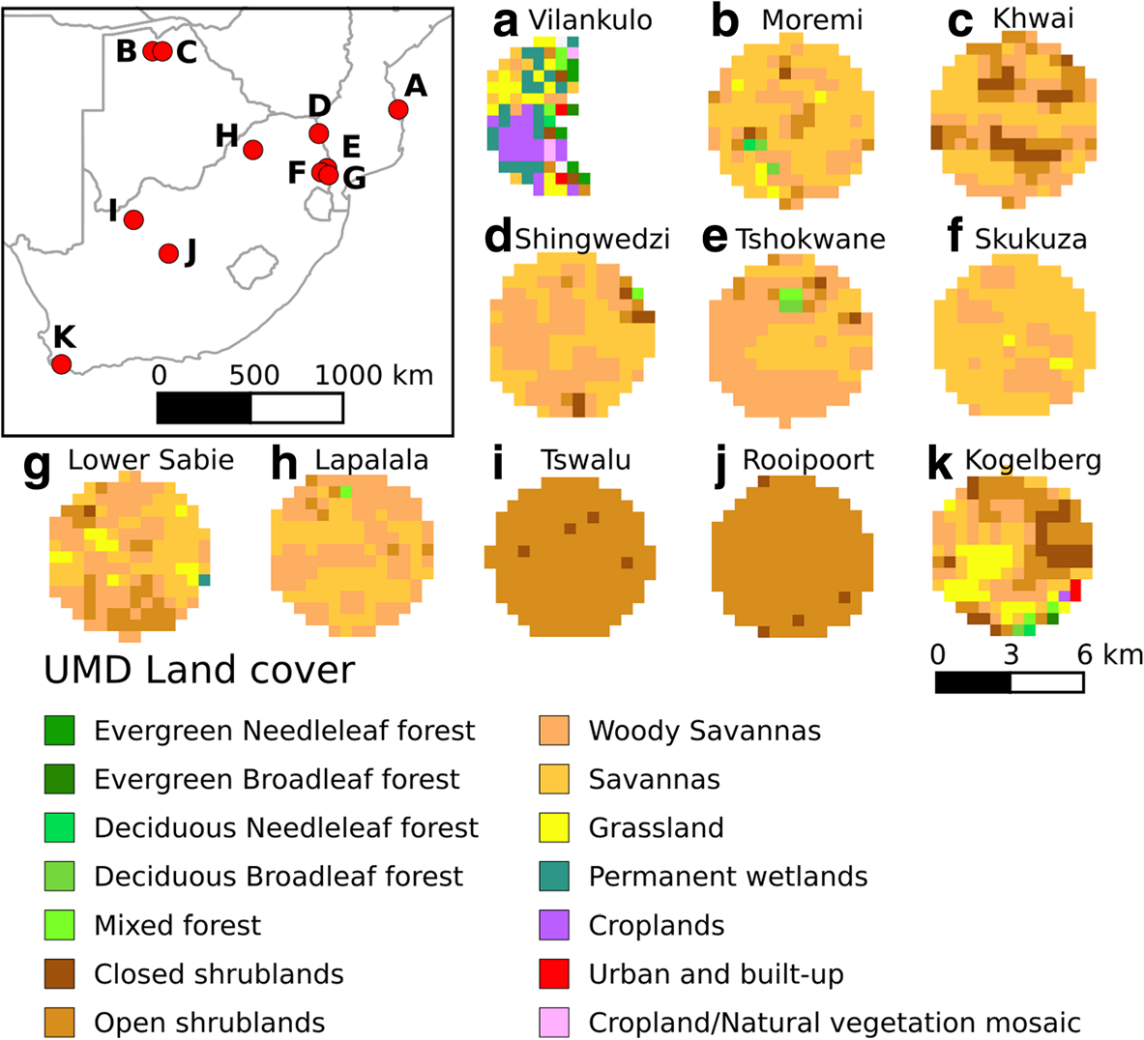
Map of collection sites and their land cover type within a 3 km radius area (**a**-**k**). The latest (2013) University of Maryland Land (UMD) cover type data was used. Snow/Ice and Barren land cover type are omitted from UMD legend as our study localities do not include any of those categories. Capital letters superimposed on the map insert correspond to equivalent small letter landcover charts

*A priori* selection of localities was aimed at sampling the widest range of biomes, land cover types and geographical spread within the available time and resource constraints, with an emphasis on South Africa, which is the focal country for studies on zoonoses by the University of Pretoria. Georeferenced locations and land-cover types of these localities are provided in Table 1 and Fig. 1, respectively. Locations where mosquitoes were captured were mainly savanna and grassland habitats, except for Vilankulo where some land cover consisted of croplands (Fig. 1).

**Table 1:** Mosquito composition and diversity data from collections performed in wildlife reserves in southern Africa 2017. Lettering in the first column corresponds to the letter in the map insert in Fig. 1

	Locality	Latitude, Longitude	Survey period	N_TT_ ± SD^a^	N_LT_ ± SD^a^	Net *vs* light trap *P*-value^b^	% vectors^c^	CCM^d^	CCM_av_^d^	CCM_NT_^d^	CCM_LT_^d^	H
	Mozambique											
A	Vilankulo	21°57.1'S, 35°18.8'E	21–28 January	784 ± 662	na	na	93.7	2353 (13) [5]	2205 (5) [3]	2353 (13) [5]	na	0.89
	Botswana											
B	Moremi Game Reserve	19°07.1'S, 23°23.2'E	20–23 February	115 ± 93	163 ± 130	< 0.001	76.2	1339 (27) [8]	1020 (7) [4]	689 (15) [6]	650 (25) [8]	1.74
C	Khwai Community Conservancy Area	19°07.3'S, 23°52.1'E	20–23 February	186 ± 128	399 ± 336	< 0.001	64.2	1356 (18) [8]	870 (4) [3]	559 (12) [4]	797 (16) [8]	1.79
	Kruger National Park, South Africa											
D	Shingwedzi	23°06.7'S, 31°27.4'E	18–21 March	17 ± 9	33 ± 10	< 0.001	23.2	168 (20) [5]	39 (6) [3]	68 (15) [4]	100 (16) [5]	2.12
E	Tshokwane	24°47.1'S, 31°51.3'E	24 March	116 ± 56	33 ± 18	< 0.001	68.4	414 (20) [6]	283 (7) [4]	348 (20) [6]	66 (7) [4]	1.71
F	Skukuza	24°59.1'S, 31°34.8'E	22–23 March	17 ± 6	19 ± 8	< 0.001	74.3	109 (14) [4]	81 (5) [3]	52 (7) [3]	57 (11) [4]	1.80
G	Lower Sabie	25°7.2'S, 31°55.6'E	25 March	46 ± 8	na	na	76.9	91 (13) [4]	70 (8) [3]	91 (13) [4]	na	1.76
	Other South Africa											
H	Lapalala Nature Reserve	23°53.5'S, 28°16.0'E	7–10 April	30 ± 20	17 ± 11	< 0.001	56.2	297 (19) [5]	2,205 (5) [3]	213 (15) [5]	84 (13) [4]	1.99
I	Tswalu Game Reserve	27°17.8'S, 22°28.1'E	14–17 February	2 ± 0	9 ± 9	0.01391	73.3	60 (6) [3]	44 (3) [2]	6 (2) [2]	54 (6) [3]	1.13
J	Rooipoort Nature Reserve	28°56.2'S, 24°10.1'E	7–11 March	115 ± 58	29 ± 20	< 0.001	62.6	1000 (13) [4]	626 (9) [2]	689 (10) [3]	311 (11) [4]	1.40
K	Kogelberg Nature Reserve	34°19.3'S, 18°58.1'E	9–11 February	66 ± 34	73 ± 36	< 0.001	96.7	695 (11) [4]	672 (3) [2]	331 (5) [2]	364 (9) [4]	0.24

*Abbreviations*: *H*, Shannon’s index, *na*, not available

^a^“N_NT_ ± SD” and “N_LT_ ± SD” denote the average number of mosquitoes captured each night in net and CDC light traps, respectively

^b^The net *vs* light trap species composition significance test results analysis

^c^“% vectors” indicates the percentage proportion of mosquitoes that are known human disease vectors out of the total number of specimens

^d^“CCM” denotes community composition measures (number of mosquito specimen captured followed by the number of unique species collected in parenthesis followed by the number of unique genera collected in square brackets) for combined net and CDC light traps (CCM), for combined net and CDC light traps of known human disease vector species, for net trap collections only (CCM_NT_) and CDC-light trap collections only (CCM_LT_)

We moved trap locations each night to cover as many different habitat types as possible and therefore get the widest possible range of species. However, within each locality, all the different trap locations clustered within an arbitrary 10 km distance (Euclidean distance) were collectively designated with one locality name (e.g. Kogelberg, Tswalu or Shingwedzi, etc.), whereas trap sites more than 10 km apart were recorded and named separately (e.g. Skukuza, Lower Sabie and Tshokwane).

### Collection methods

**Fig. 2:**
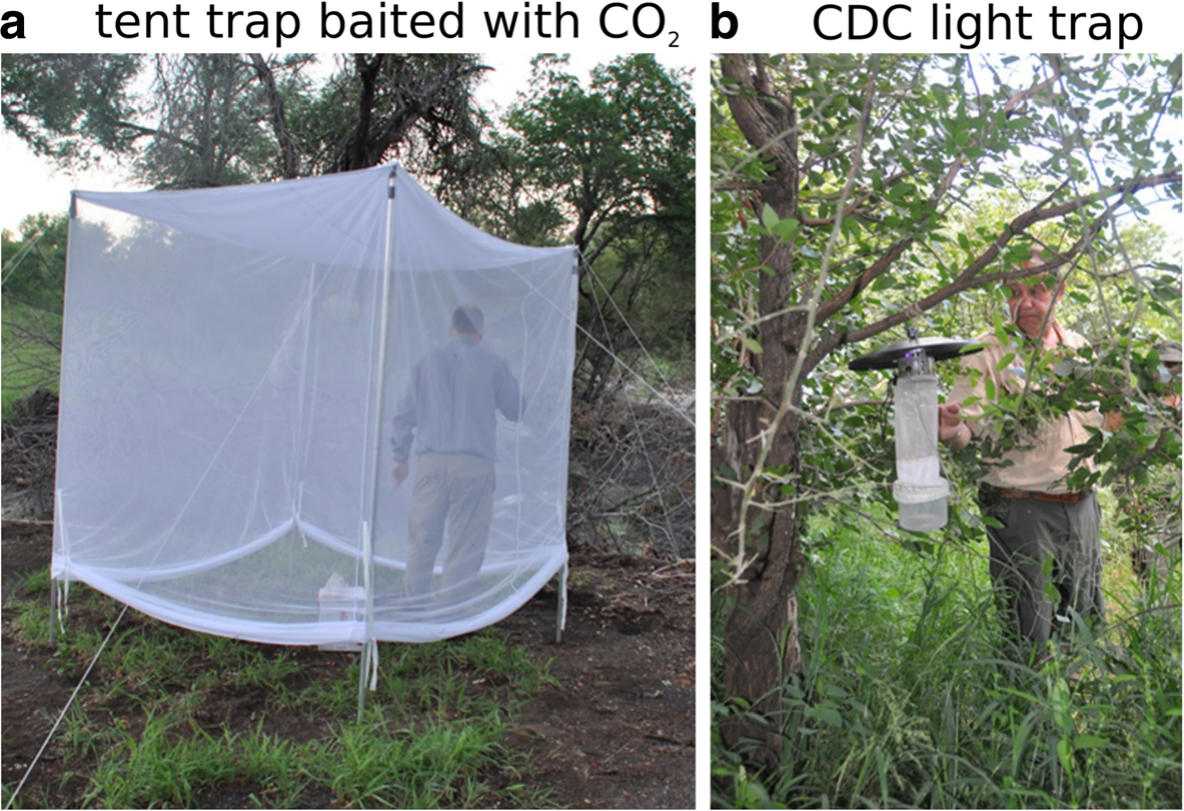
Traps used for this study. **a** Mosquito net trap baited with polystyrene box containing dry ice placed on the ground in centre of trap. **b** Center for Disease Control (CDC) light trap with white light baited with dry ice

We placed three net traps ([70], Fig. 2a) and three CDC white fluorescent light traps (Fig. 2b) each night, each baited with CO_2_ in the form of dry ice as attractant. Light traps were not deployed at the Mozambican sites, where we used CO_2_-baited net traps and also pyrethrum knockdown catches within rural dwellings (the latter results not used for analyses here, but mention is made of species collected for context). At other sites, traps were usually paired consisting of one net trap and one CDC trap that were placed within 100 m from each other, and each pair of traps was placed several hundred metres or several kilometres apart. CDC light traps were not used in Vilankulo and in Lower Sabie the CDC light traps failed because of battery charging problems and the catch nets falling off the traps.

Traps were placed and baited late afternoon, before dusk, and emptied before first light each morning. Mosquitoes were removed from within net traps using hand held aspirators and transferred into mesh-topped polystyrene cups, and the light traps by tying off the neck-sleeve of the collection bucket. All collections were killed within a few hours thereafter by freezing and immediately examined microscopically for species identification. Representative specimens were pinned as reference material and the remaining specimens grouped by species and frozen in liquid nitrogen for subsequent virus isolation assays.

Supplementary mosquito collections were performed on an *ad-hoc* basis using larval dipping for larvae and pupae, (method described in [71]), and sweep netting and pyrethrum knock down catches for adults. After capture the larvae and pupae were reared individually to adults in single tubes provided with fish flakes (Tetra-Min™- Tetra Holdings, VA, USA) as a food source. Pyrethrum knock down catches were performed in homes in Mozambique.

### Species identification methods

Morphological species identification was carried out using keys and descriptions [59, 60, 72, 73]. In cases of doubt in the morphological identifications, pinned specimens were compared with specimens in the reference collections of the South African National Institute for Communicable Diseases. For members of the *Anopheles gambiae* complex and *Anopheles funestus* group, laboratory PCR identifications were done on individually-tubed specimens preserved in tubes containing silica gel (*funestus* group) or 80% ethanol (*gambiae* complex) following protocols in [74–76], respectively. *Anopheles funestus* group members from Moremi and Khwai were not identified to group member because the DNA of these specimens was too degraded for preservation by the time we got back to the base camp.

In southern Africa, *Culex quinquefasciatus* and *Culex pipiens* do not hybridize and are easily distinguishable as adults [77]. Some adult female species cannot be reliably distinguished and in these instances they were identified to the two possible species they could be.

### Data analysis

Net and light trap mosquito species counts were averaged across all the nights of mosquitoes captured at each location. Species composition comparisons between net and light traps were done using Wilcoxon rank sum test (*P* > 0.5) using the R statistical package [78].

Pie charts depicting the percent proportions of catches represented by each of the major species (CDC light and net trap counts combined) in each location were created in Microsoft Excel (Version 2010) based on the raw capture data provided in Additional file 1: Table S1. CDC light traps were not performed in Vilankulo and Lower Sabie so the pie charts relevant to these locations represent only net trap data species compositions. All species that contributed relative percentage catches below 0.5% of the total catch were represented as “other spp.” pertaining to their specific genera in the pie charts. Color coding for each species was kept consistent for all pie charts.

We selected two measures for depicting taxonomic richness and species diversity. To reflect taxon richness we developed a simple indicator that provides a ‘one-glance catch-all’ measure of the number of individuals caught in a trap, followed by the number of species and then genera in brackets. For example, if 300 mosquitoes representing 12 species and 5 genera were caught in a particular trap then this catch is summarized as ‘300 (12) [5]’. For ease of reference in the discussion below, we refer to the simple numeric ‘catch-all’ measure as the ‘Community Composition Measure’ (CCM). As a measure of species diversity we used the Shannon’s index (H) [79], which takes into account not only the number of taxa (species in our case) but also the relative abundance in which the different species are represented in the catch. For example, a trap catch having 100 individuals made up of 2 species and each of the 2 species represented by 50 individuals will have a higher score (H = 0.693) than a trap catch of 100 mosquitoes made up of 2 species but of those, one species is represented by 90 individuals (H = 0.325). This index is commonly used in ecology to provide information about community composition reflecting not only the unique number of species but also its abundance [80].

QGIS version 2.18.4 was used for generating the land cover maps based on the University of Maryland (UMD) Year 2013 Land Cover Classifications. The 2013 UMD Land cover data was extracted from MCD12Q1 MODIS Land Cover Type product available from Land Processes Distributed Active Archive Center (https://lpdaac.usgs.gov/dataset_discovery/modis/modis_products_table/mcd12q1) using the HDF-EOS to GeoTIFF Conversion Tool (HEG) version 2.14.

Morisita-Horn index [81] was calculated as measure of species composition similarity following the recommendation by Wolda [82]. Morisita-Horn index of 0 means no similarity and its value close to 1 means high similarity in species composition. A dendrogram based on the Morista-Horn index similarity matrix was generated using Scipy python library (https://www.scipy.org/).

## Results

**Fig. 3:**
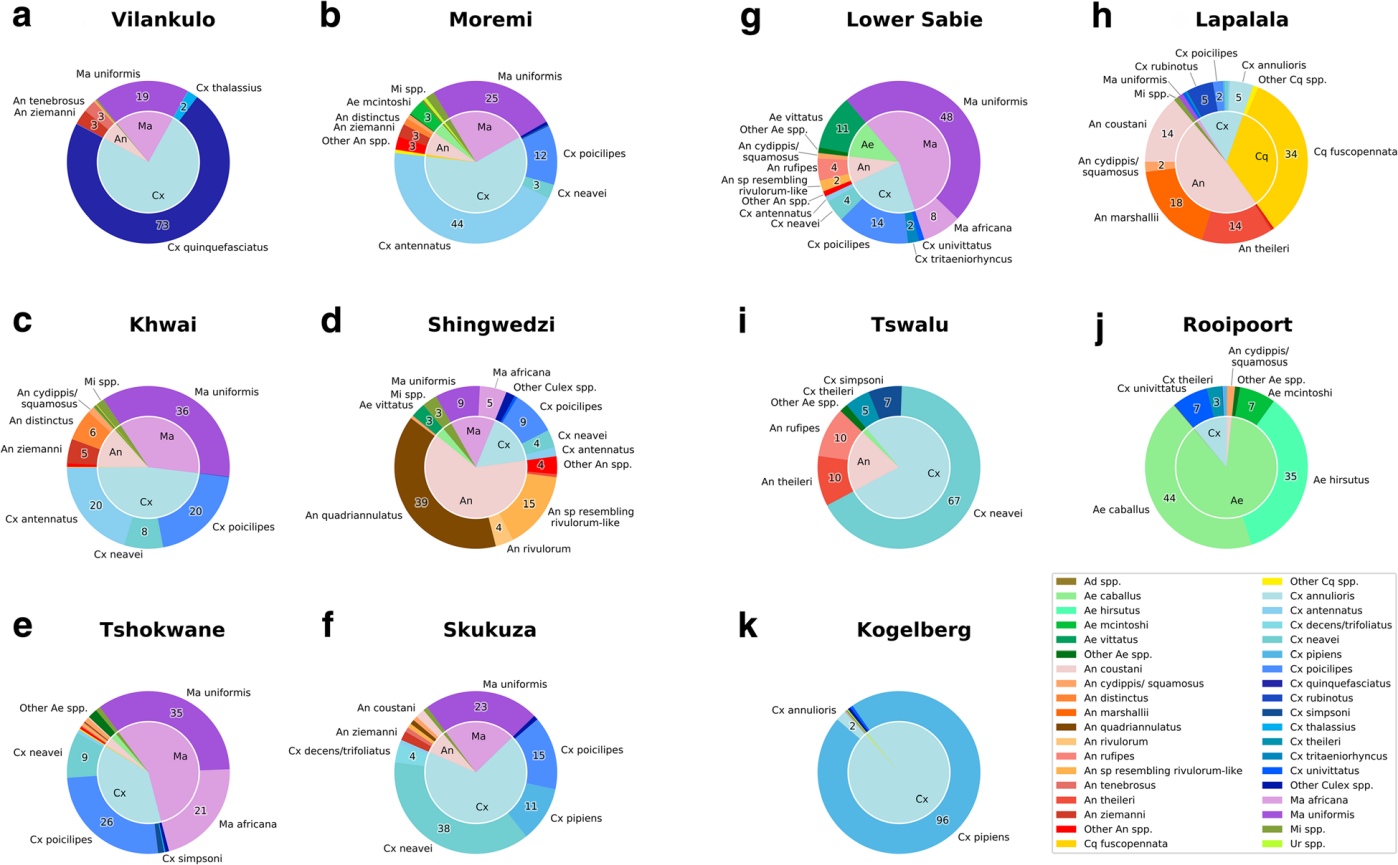
Mosquito species composition pie charts of combined net and CDC-light traps. **a** Vilankulu, MZ. **b** Moremi, BW. **c** Khwai, BW. **d** Shingwedzi, KNP, SA. **e** Tshokwane, KNP, SA. **f** Skukuza, KNP, SA. **g** Lower Sabie, KNP, SA. **h** Lapalala, Limpopo, SA. **i** Tswalu, Northern Cape, SA. **j** Rooiport, Northern Cape, SA. **k** Kokelberg, Western Cape, SA. Species with densities below 2% were grouped into respective genera as spp. Genus name abbreviations are consistent with [95]. Dark tan color was used for *Aedomyia* species; shades of greens for *Aedes*; shades of red and oranges for *Anopheles*; shades of yellow for *Coquellittidia*; shades of blue for *Culex*; shades of purple for *Mansonia*; olive green for *Mimomyia*; and lime green for *Uranotaenia*. Numbers in outer pie slices indicate proportions of individuals from total net and light trap catches within each locality. Inner pie shows the relative proportions of each genus

A total of 7882 mosquitoes were collected. Sixty-six species from 8 genera were collected in either or both the net and CDC traps and an additional 8 species, thus bringing the overall total to 74, were collected as larvae and other supplementary collections. Supplementary collections were not included in the diversity indices calculations. Additional file 1: Table S1 provides a full list of species collected, and includes a summary of geographical localities each species was found in, the total number and percentage of total catch each species comprised, the known pathogens vectored by each of the species, and reference to the publication confirming their role as a vector. Pie charts representing species composition percentages for combined net and CDC light traps are provided in Fig. 3.

In Kogelberg, a specimen keyed out as *Uranotaenia hopkinsi*, a species that according to Jupp [60] occurs in Mozambique and not in South Africa. However, morphological features such as, (i) broad band of bluish scales along eye margins, (ii) pale scales at base of wing vein R, and (iii) broad patch of bluish white scales at wing root, were noted in the mosquito from Kogelberg, which are characters that differ slightly from that described for *Ur. hopkinsi* [69].

**Fig. 4:**
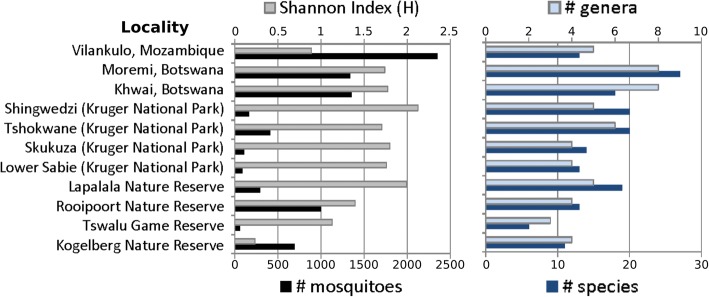
Species diversity measures for each location. Shannon’s index (top X axis) are represented in gray bars, the total number of mosquito specimens collected (bottom X axis) in black, the number of unique genera (top axis) in light blue and the total number of unique species (bottom X axis) are in dark blue

*Culex quinquefasciatus*, represented by 1708 individuals, was the most abundantly captured mosquito, but > 99% of these were collected in one locality (Vilankulo, Mozambique, see Shannon’s H in Fig. 4). *Mansonia uniformis*, with 1505 individuals captured from 8 different trapping locations, was more equitably abundant over the southern African region, followed by *Culex antennatus* (*n* = 876), *Cx. pipiens* (*n* = 689) and *Cx. poicilipes* (*n* = 592). Fourteen species were represented by only one specimen having been caught. The species found in the largest number of localities were *Anopheles squamosus*, *Cx. antennatus*, *Cx. neavei*, *Cx. poicilipes*, *Cx. univittatus* and *Mansonia uniformis*. Of all genera, *Culex* was the genus represented by the most species (*n* = 27).

Malaria vector species were collected mainly in Vilankulo, and a few in Moremi, Khwai and the Kruger National Park. However, a significant proportion of mosquitoes that vector arboviruses were collected at all locations in combined net and CDC light traps (Table 1: % vectors and CCM_av_). Their numbers of individuals comprise more than half of the total mosquito catches, except in Shingwedzi (KNP).

A significant difference in species captured between net and CDC light traps was found in all locations (Table 1, in bold print), except for in Tswalu, (Wilcoxon-rank sum test, V = 36–630, *P*-value < 0.0008). There was no significant advantage of CDC light traps over net traps in capturing higher numbers of different species (Wilcoxon-rank sum test *P*-value > 0.5). There were 9 species that were captured only in net traps and 14 species only in the CDC light traps (Table 2).

**Table 2:** Species collected in either net or CDC light traps. Species collected in multiple locations are highlighted in bold

Species only found in net traps	Species only found in light traps
*Ae. juppi* (*n* = 3)	*Ae*. *aegypti* (*n* = 1)
*Ae. ochraceus* (*n* = 8)	*Ae. fowleri* (*n* = 1)
*An. longipalpis* (*n* = 1)	*Ae. ledger* (*n* = 1)
***An. parensis*** (*n* = 3; 3 locations: Shingwedzi , Vilankulo and Lower Sabie)	*Ae. mixtus/microstictus* (*n* = 1)
*Coq. maculipennis* (*n* = 3)	*Ae. simpsoni* (*n* = 1)
***Cx. aurantapex*** (*n* = 2; 2 locations: Moremi and Tshokwane)	*Ae. subdentatus* (*n* = 1)/*Aedeomyia africana* (*n* = 1); ***Ae. furfurea*** (*n* = 10; 3 locations: Moremi, Khwai and Rooipoort)
*Cx. decens/trifoliatus* (*n* = 4)	*Cx. chorleyi* (*n* = 2)
***Cx. quinquefasciatus*** (*n* = 1708; 2 locations: Vilankulo and Rooipoort)	*Cx. duttoni* (*n* = 1)
*Cx*. *salisburiensis* (*n* = 1)	*Cx. nebulosus* (*n* = 1)
	*Cx. pulchrithorax* (*n* = 1)
	***Mi. splendens*** (*n* = 25; 3 locations: Moremi, Khwai and Shingwedzi)
	*Ur. hopkinsi* (*n* = 1)
	*Ur. mashonaensis* (*n* = 1)

The Community Composition Measure (CCM) of mosquito numbers (Table 1, Fig. 4), indicated the highest species richness (31 species in 6 genera) along the Sabie River and Tshokwane Picnic Site in the southern KNP. CCM indicated the lowest species richness at Tswalu Game Reserve in the arid Kalahari (6 species in 3 genera). The Moremi Game Reserve and Khwai Conservancy in the Okavango Delta of Botswana had fewer species than in the southern KNP but had more genera represented (29 species in 8 genera), but catch sizes were also much larger in Okavango.

With Shannon’s index greater than 1.7, Kruger National Park (KNP) and Botswana localities had greater mosquito diversities than other localities (Table 1, Fig. 4). The number of species captured in Vilankulo and Kogelberg was comparable to Kruger National Park sites but Shannon’s diversity index was lower than 1, indicating less evenness of species composition (Fig. 4). The number of species collected in Tswalu Game Reserve was lowest, but its diversity index (Shannon’s H) was higher than Vilankulo and Kogelberg, indicating the more evenness in species composition (Fig. 4). The species diversity represented in Shannon’s index (H) correlated with latitude (*R*^2^ = 0.4415, linear model *P*-value = 0.0257). Correlation between H and historic precipitation (WorldClim version 2.0) was not significant (linear model *P*-value > 0.05).

**Fig. 5:**
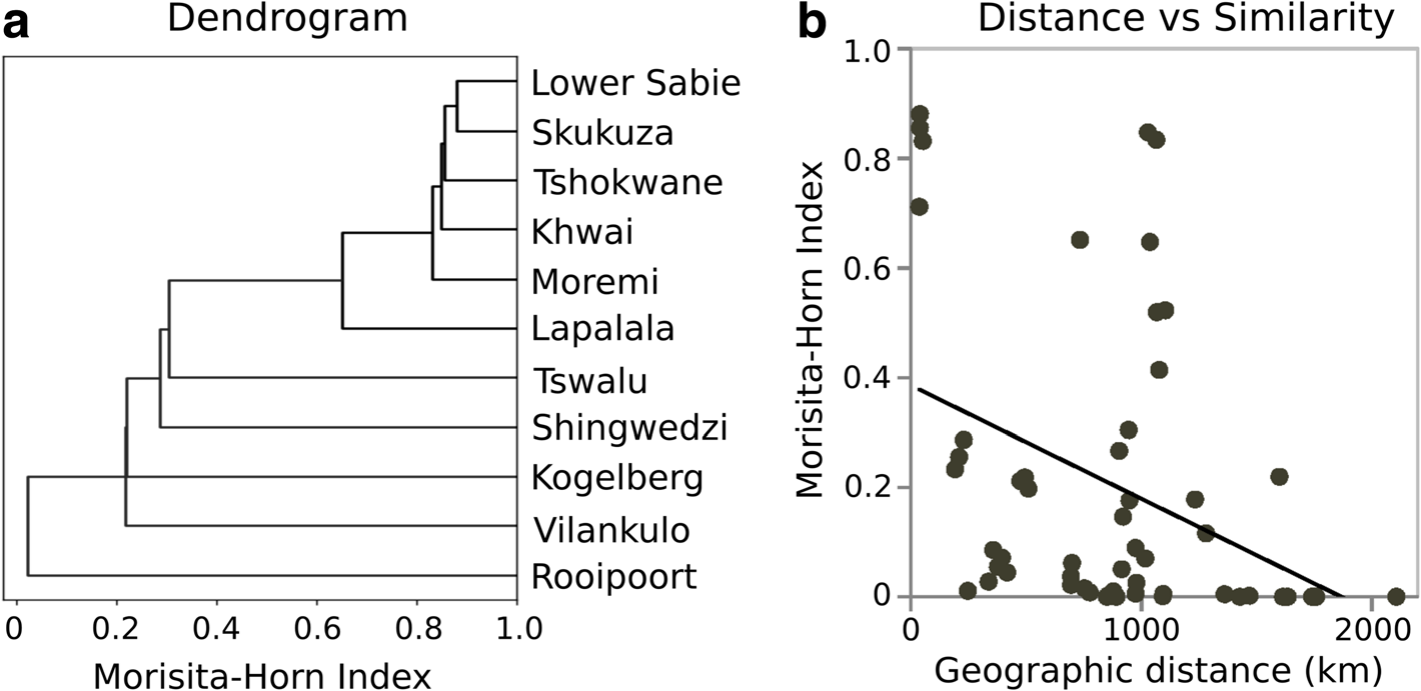
Species composition similarity. **a** Dendrogram based on the pair-wise Morisita-Horn Index, a measure of species composition similarity between any two localities. **b** Relationship between species composition similarity measured as Morisita-Horn Index and geographical distance (Linear model slope = -0.0002, *R*^2^ = 0.1399, *P*-value = 0.005)

Similarity in species composition between localities, measured as Morisita-Horn Index [81], show that southern Kruger National Park (KNP; Lower Sabie, Skukuza and Tshokwane) and Botswana (Moremi and Khwai) were similar (Morisita-Horn Index > 0.8) as shown in Fig. 5a. Generally, the species composition similarity decreased as geographical distance between two localities increased (Fig. 5b). Botswana and KNP were exceptions to this rule as they are almost 1000 km apart but yet had similar species compositions. Hence, other factors such as land cover types and availability of blood source may be affecting species composition. However, due to the limited number of sites we surveyed, we did not have enough power to detect any significant relationship between land cover types and species diversity and/or composition.

## Discussion

In a country or region where relatively little is known about mosquito populations, or presence of arboviruses, understanding the potential for arbovirus transmission is dependent on answering the following key questions “What species occur here?” and “How abundant are they and how do they vary in time and space?”. Given the potential for rapid spread of arboviruses at global scale [83, 84], and their alarming public health impacts and little capacity to prevent this [85], answers to these key questions are becoming increasingly important. These questions are even more important on the African continent where many of the current crop of emerging and re-emerging viruses have originated. Africa also hosts many relatively quiescent viruses that remain confined to sylvatic cycles.

Trapping was not done within five km of the rest camps within the wildlife reserves, to avoid collections of mosquitoes associated with human habitats. However, it should be noted that at the rest camps in Tswalu and Rooipoort nature reserves, *Ae. aegypti* and *Culex quinquefasciatus* were profound nuisances. Both of these mosquitoes are arbovirus vectors and potentially pose a risk of arbovirus transmission to guests if the viruses were inadvertently introduced.

Our CCM metric provides an intuitive sense of mosquito population attributes, that enables one to instantly get a ‘sense’ of species diversity represented in a given area (Table 1). Our trapping methods were proven to be effective in capturing potential vector species as indicated in Table 1. All locations have more than three species and two genera that are known to be arbovirus vectors (Table 1). The high numbers of one vector species, namely *Cx. pipiens* and *Cx. quinquefasciatus* (> 90%) in Kogelberg and Vilankulo, respectively, is concerning in terms of transmission potential.

Our survey also demonstrated the high degree of consistency using a combination of net traps and light traps to quickly sample mosquito species richness in a particular locality. For instance, southern Kruger National Park sites (Skukuza, Tshokwane and Lower Sabie) all had H around 1.7 (Table 1, Figs. 3 and 4) and their species compositions were very similar (Morisita-Horn Index > 0.8; Fig. 5). We collected most of the species (89.2%, of 74 species) using these two trap types and additional sampling of mosquitoes by way of collecting larvae in tree-holes, rock-pools and various other habitats, and sweep-netting through tall and dense grass along riverbanks, and conducting pyrethrum knockdown sprays in Vilankulo yielded relatively fewer species. Because of the significant differences in species captured between the net and CDC-light traps at almost all locations, we recommend using both trap types for species diversity and for outdoor biting arbovirus and malaria vector surveillance.

Despite the relative efficiency of our sampling design to survey the diversity of carbon-dioxide-attracted mosquitoes within the space of three to four days at a particular locality, clear shortfalls have also become apparent. Two years after this survey, we collected mosquitoes at Shingwedzi (KNP) in March 2017 for ten days following two months of sustained good and regular rainfall, which created excellent breeding conditions for many mosquito species. This survey yielded several additional species not found during the 2015 survey. These included *Aedes sudanensis*, *Anopheles arabiensis* and *Culex tigripes*. The reverse was also true, that despite highly favourable conditions, some species were not collected during March 2017 but were caught under very average conditions of March 2015. Furthermore, copious numbers of *An. arabiensis* were collected by Cornel, Lee and Braack, in Lapalala in February 2017, a species that had not been collected in this region for many years. There were also multiple reported cases of malaria in this region which had also been malaria free for many years previously, indicating that species distributions contract and expand periodically. This emphasises the obvious though, that a single visit even during a ‘good season’ will not yield an exhaustive catch of all species and that repeat visits in different seasons over several years are necessary to achieve a ‘complete’ picture of mosquito diversity in a region or to monitor trends in mosquito community composition.

Despite most of our sampling localities being situated in hot, summer rainfall areas, in the bushveld or savanna habitats, with abundant surface water available and similar mix of plentiful wildlife (blood meal sources) and despite the known presence of both members of the *Anopheles gambiae* complex being present in the Okavango Delta (Moremi, Khwai), and KNP, only *Anopheles arabiensis* was captured in the Okavango traps and only *Anopheles quadriannulatus* in the traps at KNP. However, larvae collected in one elephant footprint pool at Lower Sabie in southern KNP and reared to adulthood yielded a mix of *Anopheles arabiensis* and *A*. *quadriannulatus*, suggesting that the absence of one or the other species in the traps was simply an artefact of chance. The ecological determinants underpinning local dominance and abundance of these two partially sympatric species are poorly understood, with one species predominating in one region of sympatry, such as *Anopheles arabiensis* apparently more common than in the Okavango Delta than *An quadriannulatus* [86], the reverse apparently applying in the Mpumalanga Province of South Africa [87, 88], all or which likely plays a role in the presence of these species in trap catches.

Although our survey is limited in geographical and trap coverage, we found high species richness and diversity in extensive wildlife conservation areas. These areas retain historic ecological integrity and habitat diversity and are moderately or well supplied with good quality surface water for breeding substrate and have readily available sources of blood meals in the form of birds and mammals. These conditions exist in the Okavango Delta, Kruger National Park, and Lapalala Game Reserve, which all show Shannon’s indices above 1.7, but lower levels of diversity are present at localities where essential elements of favourable habitat are lacking, such as in the Kogelberg which had few medium to large birds and mammals, and Rooipoort and Tswalu Nature Reserve, which have adequate birds and mammals but very little surface water appropriate for mosquito breeding.

Subsets of the data can be usefully compared with findings of other studies. For example, Ngomane et al. [89] found that in a sample of 319 *Anopheles funestus* group mosquitoes collected from eight sites in Mpumalanga Province of South Africa between 2002–2005, 7.8% were *Anopheles funestus* (*sensu stricto*), 60.2% *An. rivulorum* (presumably includes *An. rivulorum-*like), 10.7% *An. vaneedeni*, 10.9% *An. parensis* and 10.3% *An. leesoni*. Our collective sample of 63 *Anopheles funestus* group captured over a two-week visit (includes a few individuals collected from traps not reported on here) at five collection areas in Kruger National Park comprised 77.7% specimens resembling *An. rivulorum-*like, 17.5% *An. rivulorum*, 3.2% *An. parensis* and 1.6% *An. leesoni*. These differences in species assemblage across geographically-adjoining areas are likely due to different sampling methodologies, Ngomane et al. [89] obtaining most of their specimens from night-time human landing catches and day-time catches of mosquitoes resting in natural shelters, compared to our net trap and light trap techniques. These differences also emphasize the need to standardize trapping techniques to allow for valid comparisons.

Steyn et al. [90] spent 15 days sampling mosquitoes at multiple sites along the Limpopo River valley, covering some 300 miles from Vaalwater (very close to Lapalala Game Reserve) eastwards to the northern Kruger National Park at Pafuri (not far from Shingwedzi). Similar to our sampling surveys at Lapalala and Shingwedzi, their survey was also in March in late wet-season. Their survey yielded 538 mosquitoes comprising 21 species in three genera. Their catches are of the same general scale as ours, where we record 20 species (5 genera) at Shingwedzi and 19 (5 genera) at Lapalala in much shorter collection periods. Steyn et al. [90] based their publication mostly on larval collections (409 larvae) from tree-holes, pools at quarries and borrow-pits, rock pools and even large snail shells, supplemented with adult catches (129). Importantly however, despite the similarity in species richness, nearly 50% of the species they caught or reared were different to those in our collections, with a predominance of *Aedes* species as can be expected from the types of breeding sites they sampled. Once again, the need to standardize collection techniques is emphasized. Long term monitoring of mosquito populations and comparisons between different sites are reliant on quick efficient trapping techniques directed at sampling important species, whilst ecological studies aimed at understanding species richness will require a wide a range of collection methods.

The Shingwedzi River and Sabie River collection areas roughly bisect the northern and southern regions of the Kruger National Park in South Africa. The habitat along these rivers is fairly representative of these two regions, especially as it relates to mosquito breeding site types. In March 2015, we collected 20 species (5 genera) in the north and 31 species (6 genera) in the south of KNP. In April 1953, Schultz et al. [91] surveyed culicine mosquitoes by way of larval collections throughout the KNP, and collected a total of 907 mosquitoes (799 larvae, 108 adults) made up of 25 species in four genera; three species were *Anopheles*, one *Orthopodomyia*, 12 *Aedes* and nine *Culex*. Schultz et al. [91] collected 12 species and one genus (*Orthopodomyia*) over 18 days that we did not collect in our stay in eight days. Conversely, we collected 15 species and three genera (*Coquellittidia*, *Mansonia* and *Mimomyia*) that they did not collect. Clearly each collection method has its own limitations and there is a need to clearly identify the purpose of the survey and to design appropriate sampling strategies to optimize appropriately targeted outcomes, especially if time and manpower are limited. Our combination of net traps and light traps proved efficient in yielding a good range of species and genera in very limited time, and were good at collecting a wide range of *Culex* and *Anopheles* species not well represented in the Schultz et al. [91] and Steyn et al. [90] collections, but were poor at trapping *Aedes* in comparison with the larval collections in the Steyn et al. [90] and Schultz et al. [91] collections.

To give some sense of what kind of species richness can be expected after very intensive and continuous sampling; van der Linde et al. [92] placed four light traps at weekly intervals for three years in a rural area near Bloemfontein in the central region of South Africa. They collected 143,438 mosquitoes representing 25 species in four genera, of which 85% were of the three species *Aedes juppi*, *Aedes durbanensis* and *Culex theileri*. Our Rooipoort site is within a couple of hundred kilometres from Bloemfontein, and we collected 1000 mosquitoes representing 13 species in 4 genera during five days of sampling using net traps and light traps. This again suggests that even relatively short sampling periods of three to five days using CO_2_ baited net traps and light traps can be effective in providing broad insight to species composition and abundance if done at the appropriate seasonal time.

## Conclusions

For disease epidemiological or surveillance aimed at collecting mosquitoes of as broad a range of species as quickly as possible, our findings suggest that a combination of night-operated CO_2_ baited net traps and light traps provides good representation of mosquito diversity and abundance in an area even during relatively short sampling visits lasting 3–5 nights. *Anopheles*, *Culex*, *Mansonia*, *Coquellittidia* and *Mimomyia* are well represented in our collections, but *Aedes* appeared to be under-represented. This may be because many *Aedes* are predominantly day-biting and, at least in some cases, also have shorter periods of seasonal abundance. For *Aedes* collections it is therefore probably better to do larval collections from container habitats, supplemented with day-operated odour-baited BG traps or similar devices [93]. We also find a simple Community Composition Measure (CCM) which combines numbers of mosquitoes captured, number of species, and number of genera, a far more useful indicator of mosquito community status and structure at a particular sampling site than the Shannon’s index, although the two measures do complement each other and together provide a more informed assessment. Limited time and resources constrained our ability to develop a finer-grained understanding of mosquito communities across South Africa, yet the selected broad coverage of the sites represented in this study does provide good initial insight as to where high diversity and numbers are likely to be found, such as in the extensive untransformed conservation areas located in warm climates and having a combination of diverse mammals and birds as blood meal source and good stands of surface water of different types; arid environments and areas poor in birds and animals appear to support lower species richness. This may seem intuitively obvious, but is useful confirmation.

## Additional file

Additional file 1: **Table S1.** Mosquito species capture data and disease vector status for each of the mosquito species captured. (XLSX 31 kb)

## Abbreviations

BAGV: Bagaza virus
BANV: Banzi virus
BATV: Batai virus
BBKV: Babanki virus
BUNV: Bunyamwera virus
BWAV: Bwambwa virus
CCM: Community Composition Measure
CDC: Centers for Disease Control and Prevention
CHIKV: Chikungunya virus
CYV: Chaoyang virus
DENV1-4: Dengue virus serotypes 1 to 4
GERV: Germiston virus
H: Shannon Index
KNP: Kruger National Park
MIDV: Middelburg virus
NDUV: Ndumu virus
NRIV: Ngari virus
ONNV: O’Nyong-Nyong virus
PGAV: Pongola virus
QBV: Quang Binh virus
RVFV: Rift Valley fever virus
SFV: Semliki Forest virus
SHUV: Shuni virus
SINV: Sindbis virus
SPOV: Spondweni virus
UGSV: Uganda S virus
USUV: Usutu virus
WESV: Wesselsbron virus
WITV: Witwatersrand virus
WNV: West Nile virus
YFV: Yellow fever virus
ZIKV: Zika virus
